# Comparative Evaluation of Commercial Protein A Membranes for the Rapid Purification of Antibodies

**DOI:** 10.3390/membranes13050511

**Published:** 2023-05-12

**Authors:** Joshua Osuofa, Scott M. Husson

**Affiliations:** Department of Chemical and Biomolecular Engineering, Clemson University, Clemson, SC 29634, USA

**Keywords:** Protein A, membrane chromatography, dynamic binding capacity, elution volume, permeability, bioprocessing industry, monoclonal antibody, impurity clearance

## Abstract

Protein A chromatography is ubiquitous to antibody purification. The high specificity of Protein A for binding the Fc-region of antibodies and related products enables unmatched clearance of process impurities like host cell proteins, DNA, and virus particles. A recent development is the commercialization of research-scale Protein A membrane chromatography products that can perform capture step purification with short residence times (RT) on the order of seconds. This study investigates process-relevant performance and physical properties of four Protein A membranes: Purilogics Purexa™ PrA, Gore^®^ Protein Capture Device, Cytiva HiTrap™ Fibro PrismA, and Sartorius Sartobind^®^ Protein A. Performance metrics include dynamic binding capacity, equilibrium binding capacity, regeneration-reuse, impurity clearance, and elution volumes. Physical properties include permeability, pore diameter, specific surface area, and dead volume. Key results indicate that all membranes except the Gore^®^ Protein Capture Device operate with flow rate-independent binding capacities; the Purilogics Purexa™ PrA and Cytiva HiTrap Fibro™ PrismA have binding capacities on par with resins, with orders of magnitude faster throughput; and dead volume and hydrodynamics play major roles in elution behavior. Results from this study will enable bioprocess scientists to understand the ways that Protein A membranes can fit into their antibody process development strategies.

## 1. Introduction

The specificity of Protein A to the Fc region of monoclonal antibodies and related products that contain an Fc binding domain has allowed Protein A chromatography to become a platform technology in the bioprocessing industry since the 1980s [1,2]. This specificity leads to high product purity in the first purification step. Despite having high specificity towards the product, Protein A chromatography using resin columns has low productivity (defined as rate of protein produced per column volume). The root cause is that binding capacity decreases as flow rate increases. Continuous chromatography methods, such as periodic counter current chromatography, simulated moving bed, and sequential multicolumn continuous chromatography, can increase productivity compared to batch chromatography [3,4,5,6]. However, there is much room for improvement by replacing resin columns with membrane chromatography columns that can achieve high capacities using orders of magnitude shorter residence times as a result of large pores that enable convective transport of protein molecules to binding sites. The significance of this convective transport is that binding capacities in membrane adsorbers typically do not depend much on flow rate. At process scale, fast operating speed already has led to widespread adoption of membrane adsorbers for polishing step purifications [7,8].

With resin chromatography being a mature technology, many studies have comparatively characterized the performance and physical characteristics of Protein A resins [9,10,11,12,13,14]. Most recently, Pabst et al. evaluated the characteristics of 12 Protein A resins to provide a benchmark for users and developers.

On the other hand, far fewer studies have evaluated Protein A membrane chromatography, mainly because the technology is less commercially mature. However, within the last five years, research-scale Protein A membrane chromatography products have emerged from a few companies. These include Purexa™ PrA from Purilogics, Fibro™ PrismA from Cytiva, Protein Capture Device from Gore^®^, and Sartobind^®^ Protein A from Sartorius. The objective of this study was to evaluate the performance of these research-scale Protein A membrane products and compare their performance and characteristics. The study serves as a guide for deeper understanding of Protein A membrane technology and how it differs from resin technology, as a benchmark for future Protein A chromatography technologies, and as a tool for academic and industrial scientists to understand how Protein A membranes can fit into their process development strategies.

## 2. Materials and Methods

### 2.1. Materials

Protein A stationary phases included Purilogics Purexa™ PrA (0.2 mL, product no. SCANX12, Greenville, SC, USA), Cytiva HiTrap Fibro™ PrismA (0.4 mL, product no. 17549856, Marlborough, MA, USA), Gore^®^ Protein Capture Device (1.0 mL, product no. PROA101, Newark, DE, USA), Sartorius Sartobind^®^ Protein A (2.0 mL, item no. 93PRAP06HB-12—A, Göttingen, Germany). Assays included Amersham^®^ HCPQuant CHO Kit from Cytiva (Product no. 29496737, 96-well plate format, Marlborough, MA, USA) and Qubit™ dsDNA Broad Range Assay kit from Invitrogen™ (Catalog no. Q32853, Waltham, MA, USA). The following chemicals were purchased from MilliporeSigma (Burlington, MA, USA) with purities given in wt%: Phosphate buffered saline powder (1X PBS packets), citric acid (99%), trisodium citrate dihydrate (99%), sodium hydroxide (98%), and acetone (99.5%). Polyclonal human immunoglobulin (hIgG) was purchased from Lee BioSolutions (Maryland Heights, MO, USA).

### 2.2. Physical Properties

#### 2.2.1. Surface Area Measurements

Surface area measurements were performed by Brunauer–Emmett–Teller (BET) nitrogen adsorption using a Micromeritics Gemini 2360 (Norcross, GA, USA). Membranes were cooled to −196.15 °C. Nitrogen was added into the sample chamber in controlled increments until the pressure equilibrated. The amount of adsorbed nitrogen was measured by the instrument, and the surface area was calculated by the Micromeritics Gemini 2360 V1.03 software.

#### 2.2.2. Pore Diameter Measurements

A capillary flow porometer (CFP-34RTG8A-X-L4; PMI, Inc., Ithaca, NY, USA) was used to determine the average pore diameter of the membranes via a fluid displacement technique. Membrane pores were wetted with Galwick™ wetting fluid. Galwick™ was displaced by gradual increases in pressure due to increasing air flow rate. For reference, the pressures and flow rates were measured for the membrane in the dry state. Capwin Control software v6.71 was used to calculate the mean pore diameter using Equation (1), where P is the pressure required to displace the wetting liquid from the pore, γ is the surface tension of the liquid (16 dynes/cm for Galwick™), D is the pore diameter, and θ is the contact angle between the liquid and pore wall.

P = 4γcos(θ)/D(1)

#### 2.2.3. Permeability and Pressure Drop Measurements

A syringe pump with a 50 mL syringe was used to flow 1X PBS pH 7.4 buffer through each membrane device. A Heise 3084 digital pressure gauge was placed between the pump and the membrane to measure the generated back pressure. At each flow rate, the equilibrium pressure was recorded. The measurement was repeated three times at each flow rate, and flow rates were varied to achieve residence times from 5 to 120 s. For each membrane, the thickness was calculated from manufacturer stated volume and diameter. Permeability was calculated from the pressure versus flow rate data, according to Equation (2), where k is permeability (cm^2^), u is linear velocity (cm/h), µ is viscosity of water (bar h), L is membrane thickness (cm), and ΔP is pressure (bar) [15].

k = µLu/ΔP(2)

### 2.3. Performance Measurements

#### 2.3.1. Buffer Preparation

Loading buffer (B1), 1X PBS at pH 7.4, was prepared by dissolving one PBS buffer packet in 1 L of deionized water. Elution buffer (E1), 0.1 M citric acid at pH 3.0, was prepared by mixing 0.1 M citric acid with 0.1 M trisodium citrate until the desired pH value of 3.0 was reached. Clean-in-place (CIP) was performed using 0.1 M NaOH.

#### 2.3.2. Breakthrough Behavior and Dynamic Binding Capacity (DBC_10_) Measurements

DBC_10_ measurements were performed using an AKTA Purifier 100 with Unicorn software (5.31) from Cytiva. Table 1 lists the steps in the chromatography procedure. Polyclonal human IgG (hIgG) solutions were prepared by dissolving hIgG in buffer B1 and subsequently filtering through a 0.2 µm cellulose acetate filter prior to use. Two loading concentrations were used: 2 mg/mL hIgG and 5 mg/mL hIgG. hIgG was loaded to column saturation. Table 2 summarizes the flow rates used for experiments with 5 mg/mL hIgG. Experiments with 2 mg/mL hIgG were performed at 12 and 120 s residence time. DBC_10_ values were calculated at 10% breakthrough using Equation (3) and represent an average of at least three measurements. In Equation (3), DBC_10_ is the dynamic binding capacity at 10% breakthrough (mg protein/mL column volume), V_break_ is the effluent volume (mL) at 10% breakthrough, V_dead_ is the dead volume of the system (mL), C_o_ is the feed concentration (mg protein/mL solution), and V_col_ is the stationary phase column volume (mL).

DBC_10_ = (V_break_ − V_dead_)C_o_/V_col_ (3)

#### 2.3.3. Static Mode Equilibrium Binding Capacity Measurements and Application of the Langmuir Adsorption Isotherm

Protein binding studies were conducted in batch mode until equilibrium was reached between protein in solution and adsorbed protein. For Purilogics Purexa™ PrA and Sartorius Sartobind^®^ Protein A, membranes were soaked in hIgG solutions and placed on a shaker (r.t., 120 rpm) for 12 h. The initial and final protein concentrations were measured using a Thermo Fisher Nanodrop™ One UV-VIS Spectrophotometer. A calibration curve of hIgG concentration in solution was prepared against measured hIgG concentrations from the Nanodrop device. Initial hIgG concentrations ranged from 0.2 to 5 mg/mL. Membrane to solution volume ratio was kept constant for each concentration. The equilibrium binding capacity was calculated by a mass balance Equation (4) and is reported as the mass of adsorbed protein per volume of membrane. In Equation (4), SBC is the static binding capacity (mg protein/mL column volume), q is the protein adsorbed on the stationary phase (mg protein/mL column volume), C_o_ is the feed concentration (mg protein/mL solution), C_eq_ is the equilibrium protein concentration (mg protein/mL solution), V_col_ is the stationary phase column volume (mL), and V_sol_ is the volume of protein solution (mL). Langmuir adsorption isotherm parameters (Q_max_, K_d_) and their standard errors were fitted using the *fitnlm* command in MATLAB software version R2022a using Equation (5). In Equation (5), q_max_ is the maximum binding capacity (mg protein/mL membrane) and K_d_ is the Langmuir apparent dissociation equilibrium constant (mg/mL).

SBC = q = (C_o_ − C_eq_)V_sol_/V_col_ (4)

q = q_max_C_eq_/(K_d_ + C_eq_) (5)

#### 2.3.4. Dynamic Mode Equilibrium Binding Capacity Measurements

For all membrane adsorbers, equilibrium binding capacity studies were performed in dynamic mode at a residence time of 120 s using an AKTA Purifier 100. Load concentration was 5 mg/mL hIgG. The hIgG solution was loaded onto the device to column saturation. Elution was performed using 0.1 M citric acid at pH 3.0. The elution peak was collected using an AKTA Frac-900 fraction collector. The hIgG concentration in the elution peak was measured using the Thermo Fisher Nanodrop™ One UV-VIS Spectrophotometer, and the solution mass was measured gravimetrically. The equilibrium binding capacity was calculated as the quotient of eluted mass of protein and volume of membrane as shown in Equation (6). M_p_ is the mass of protein in the elution peak (mg), and V_col_ is the stationary phase column volume (mL).

q = M_p_/V_col_ (6)

#### 2.3.5. Elution Volume (EV)

Protein elution was studied using membranes that were loaded to saturation with 2 mg/mL hIgG and eluted using E1 buffer. Two residence times were tested: 12 and 120 s. To standardize the measurement, the beginning of the elution peak was defined as the point where absorbance reached 100 mAU at 280 nm wavelength, and the end of the elution peak was defined as the point where absorbance fell below 100 mAU. Runs were performed in duplicate for each membrane. To assess tailing in the elution peaks, tailing ratio was calculated using Equation (7), where EV is elution volume as measured above, and f is the distance from the leading edge to the midpoint of the peak.

T = EV/2f(7)

#### 2.3.6. Clean-in-Place (CIP) Study

Ten consecutive DBC_10_ measurements were performed on three of the Protein A devices. Residence time was set to 30 s for all steps (load, wash, elute, and CIP). hIgG concentration was 5 mg/mL in 1X PBS pH 7.4. The elution buffer was 0.1 M citric acid pH 3. CIP was performed by flowing two column volumes of 0.1 M NaOH through the column after each run. The coefficient of variance for DBC_10_ over the 10 runs was evaluated using Equation (8), where CF is the coefficient of variation, σ is the standard deviation of V_break_ over 10 runs, and µ is the mean V_break_ over 10 runs.

CF = σ/µ(8)

#### 2.3.7. Capture from Clarified Cell Culture Harvest

AntiHIV IgG1 mAb was expressed and purified according to the protocol given in Klaubert et al. [16]. Table 3 lists the biophysical characteristics of the IgG1 mAb. Isoelectric point (pI) and molecular weight were calculated from the amino acid sequence using the Expasy ProtParam online tool (https://web.expasy.org/protparam, accessed on 1 November 2021). Cell culture harvest was filtered through 0.2 µm cellulose acetate filters prior to loading onto membrane columns. Membrane columns were loaded to approximately 40% of the DBC_10_ at 12 s residence time. Equilibration, elution, and wash steps were conducted at 12 s residence time in the same way as listed in Table 1 for hIgG experiments. The following metrics were evaluated for all membrane columns: mAb yield, EV, HCP clearance, and DNA clearance. Yield was calculated as the quotient of mAb mass in the elution pool and mAb mass in the filtered feed. mAb masses were measured using a Roche Cedex Bioanalyzer (Indianapolis, IN, USA). EV was measured as described in Section 2.3.5. HCP clearance was performed by ELISA assay using an Amersham™ HCPQuant CHO Kit from Cytiva (Marlborough, MA, USA). Host cell DNA clearance was determined using an Invitrogen™ Qubit™ dsDNA Broad Range Assay kit on a Qubit 2.0 Fluorometer. Log reduction values (LRVs) were calculated by subtracting log(elution pool) from log(feed).

## 3. Results

### 3.1. Breakthrough Behavior and Dynamic Binding Capacity

**Figure 1:**
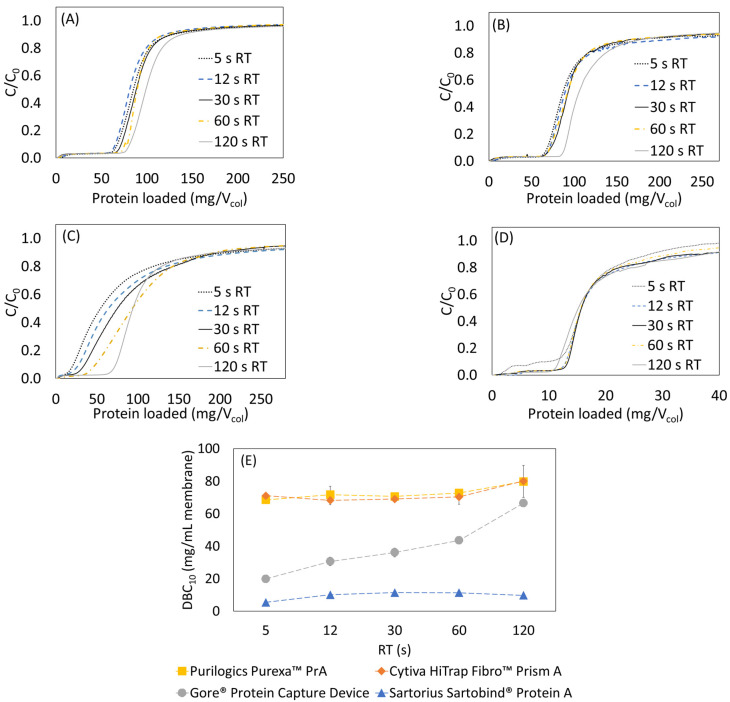
Normalized breakthrough curves from loading hIgG at 5 mg/mL onto Protein A membranes. Flow rate was varied from 5 to 120 s residence time for all stationary phases. (**A**) Cytiva HiTrap Fibro™ PrismA, (**B**) Purilogics Purexa™ PrA, (**C**) Gore^®^ Protein Capture Device, (**D**) Sartorius Sartobind^®^ Protein A, (**E**) summary of measured DBC_10_ for all stationary phases. (The dotted lines are guides to the eye).

Initial dynamic binding capacity experiments were performed using 5 mg/mL hIgG. Figure 1A–D shows the resulting breakthrough curves. Analysis of the breakthrough curves can indicate the dominant transport mechanism in each device. For the Purilogics Purexa™ PrA and Cytiva HiTrap Fibro™ PrismA membranes, the breakthrough curves from 5 to 60 s residence time were nearly overlapping, which is characteristic of convective transport of proteins through the large membrane pores to binding sites [17,18,19,20]. The DBC_10_ values were 71.0 ± 1.8 mg/mL and 69.7 ± 1.3 mg/mL. The Sartobind^®^ Protein A device also showed flow rate independent breakthrough from 12 to 60 s residence time. It had the lowest DBC_10_ of 9.6 ± 2.8 mg/mL and did experience early breakthrough at 5 s residence time.

Of the devices tested, the Gore^®^ Protein Capture Device, showed the greatest variation in performance with flow rate. The dynamic binding capacity increases by 119% from 19.9 to 43.6 mg/mL by increasing residence time from 5 to 60 s. Flow rate-dependent binding implies that pore diffusion is the limiting rate of transport. A recent study of current Protein A resin beads [13] estimated that the pore radius can vary between 30 and 60 nm, and the particle diameter can vary between 38 and 116 µm, accounting for standard deviation. According to the patent literature, the Gore^®^ Protein Capture Device utilizes embedded porous silica particles with a particle diameter of 16–24 µm and pore diameter of 100 nm [21]. Although the small particle diameter reduces the average length of diffusion, the breakthrough data suggest that the Protein A ligand is immobilized in the pores of the silica beads. Minutes of residence time are required to overcome the time scale for diffusion, similar to the limitation observed with Protein A resin beads.

The measured DBC_10_ values are comparable to state-of-the-art Protein A resin beads like the MabSelect PrismA that has DBC_10_ values of about 80 mg/mL resin with 6 min residence time using hIgG as feed [13,22]. Remarkably, the research scale Purilogics Purexa™ PrA and Cytiva HiTrap™ Fibro PrismA membrane columns can reach 70 mg/mL DBC_10_ at 5 s residence time. These devices represent breakthrough technologies for the rapid capture step purification of mAbs and related products.

Further analysis of breakthrough curves highlights the impacts of flow distribution and loading concentration on DBC_10_. Figure 1A,B for Cytiva HiTrap™ Fibro PrismA and Purilogics Purexa™ PrA membranes shows an unexpected increase in breakthrough capacity at 120 s residence time. Given that the breakthrough behavior from 5 to 60 s residence time is consistent with convection dominated transport, the observed increase in capacity at 120 s residence time likely is due to better flow distribution in the membrane device, as observed in acetone tracer experiments where sharper peaks are observed at longer residence times (Figure S2 in Supplementary Materials).

**Figure 2:**
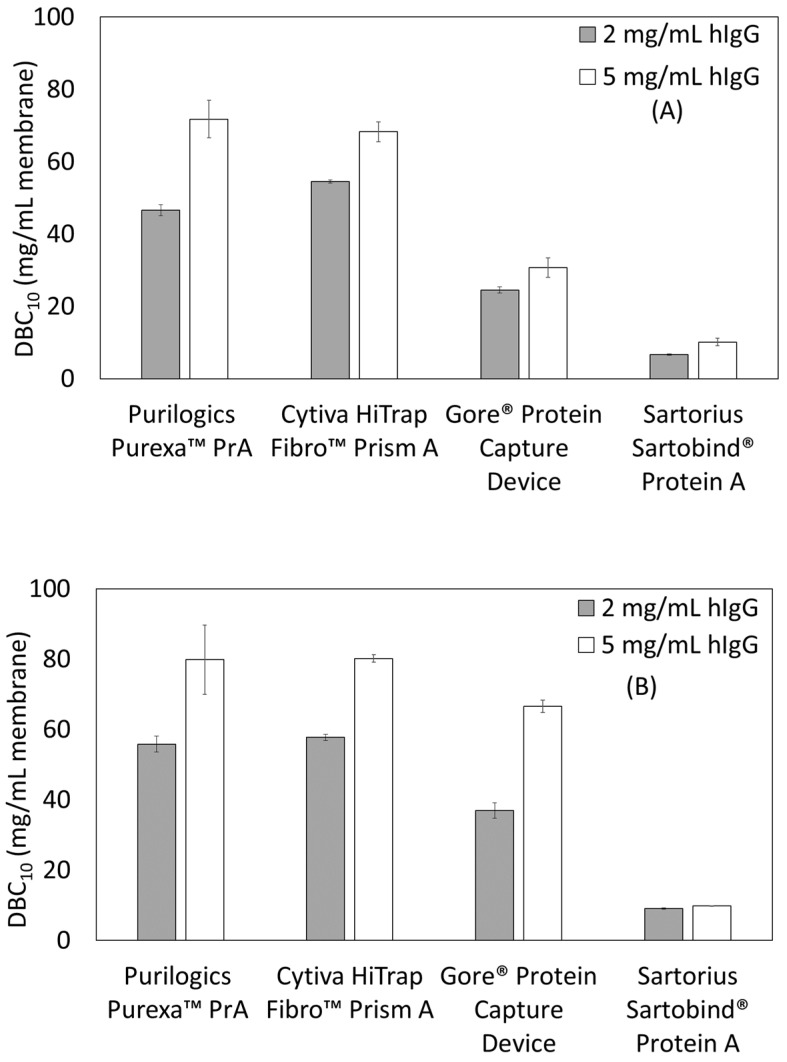
Dynamic binding capacity (DBC_10_) as a function of load concentration and residence time. (**A**) Residence time of 12 s, (**B**) residence time of 120 s.

Figure 2A,B shows the effect of feed concentration on DBC_10_. For all devices, there is an increase in capacity as concentration increases. The Purilogics Purexa™ PrA and Sartorius Sartobind^®^ Protein A membranes experience >50% increase in capacity when loading concentration increases from 2 to 5 mg/mL hIgG at 12 s residence time, while the Cytiva HiTrap™ Fibro PrismA and Gore^®^ Protein Capture Device experience approximately 25% increase. At 120 s residence time, the Sartorius Sartobind^®^ Protein A membrane does not show a significant increase in DBC_10_ while Cytiva HiTrap™ Fibro PrismA and Purilogics Purexa™ PrA show ~40% increase. The Gore^®^ Protein Capture Device shows the highest increase (80%) with respect to changing load concentration.

The reasons for protein-dependent binding behavior may differ for all membranes tested. For membranes like Purilogics Purexa™ PrA and Cytiva HiTrap™ Fibro PrismA with high dead volume, feed dilution at the inlet may be significant enough to lower the feed concentration into the linear region of Langmuir isotherm. This could result in concentration-dependent breakthrough behavior without affecting the mass transfer significantly. Similar behavior has been observed for other affinity and ion-exchange membrane adsorbers. Grunberg et al. showed that for Convecdiff Protein A membranes from Sartorius, DBC_10_ increases at higher titers [23,24]. No explanation was given for the behavior. For ion-exchange membranes, similar behavior was found and explained with the hypothesis that higher concentration improves access to ligands by overcoming film diffusion in membrane pores and combating competitive phosphate binding to ligands [25,26,27].

For Gore^®^ Protein Capture Device, which has low dead volume, the concentration dependent binding may be attributed to diffusion related transport. Similar behavior has been observed for Protein A resins. Natarajan et al. [28] evaluated the effect of feed concentration on DBC of Prosep^®^ Ultra Plus columns, and found higher DBC at higher feed concentrations, but only for longer residence times. This concentration dependent DBC behavior was explained as a result of non-equilibrium mass transfer effects, i.e., there is a maximum in DBC when mass transfer is controlled by surface diffusion rather than pore diffusion. Other studies also have found increasing DBC at higher feed concentrations for different Protein A resins [29,30].

The effect of load concentration reported for these chromatography stationary phases indicates that it is an important parameter in determining DBC values for affinity membranes. Furthermore, for future process robustness and productivity determinations, load concentration is an important parameter to consider. Further insight into the exact mass transfer or dispersive causes of concentration dependent binding may be obtained by building computational fluid dynamics models of protein concentration profiles in the membrane adsorbers. Such analysis is beyond the scope of this study.

### 3.2. Equilibrium Binding Capacity Measured in Static and Dynamic Modes

Static binding capacity experiments were performed and data were fit to the Langmuir isotherm model to determine the maximum binding capacity (q_max_) and the apparent dissociation equilibrium constant (K_d_) describing protein adsorption. For the Purilogics Purexa™ PrA and Sartobind^®^ Protein A membranes, these parameters were determined in batch mode. For the Cytiva HiTrap™ Fibro PrismA device and the Gore^®^ Protein Capture Device, binding capacity studies could not be performed in batch mode. For the Cytiva HiTrap™ Fibro PrismA device, the electrospun membranes were brittle and immediately lost shape upon opening the device. It was not possible to get an accurate measurement of the membrane volume. For the Gore^®^ Protein Capture Device, we observed much lower binding capacity than expected in static adsorption mode, when compared with DBC results (Figure S1 in Supplementary Materials). One possible explanation is that poor wettability of the PTFE membrane, noted in academic literature, may increase film resistance for adsorption and require pressure driven flow for protein adsorption [31,32]. For all other investigated membrane adsorbers, the hIgG adsorption kinetics showed typical exponential behavior as reported in other studies involving Protein A membranes and resins [33,34].

**Figure 3:**
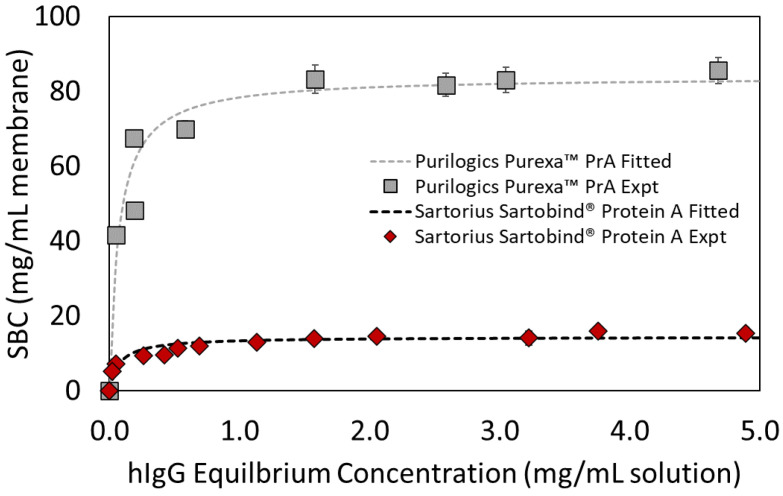
Langmuir adsorption isotherms for Purilogics Purexa™ PrA and Sartorius Sartobind^®^ Protein A membranes. Symbols represent experimental data and the curves represent fitted data with Langmuir adsorption parameters. Langmuir adsorption parameters are listed in Table 4 for both membrane devices.

Figure 3 shows adsorption isotherms for Purilogics Purexa™ PrA and Sartobind^®^ Protein A membranes, and Table 4 reports the fitted Langmuir isotherm parameters. The K_d_ values suggest that the membranes have strong affinities for the Fc-region of hIgG and are typical of Protein A affinity devices evaluated in the literature. Boi et al. [33] reported a K_d_ value of 9.34 × 10^−2^ mg/mL for a recently developed Protein A membrane. Utilizing polyclonal hIgG, Hahn et al. [10] compared Protein A resins and reported K_d_ values between 4.5 × 10^−2^ mg/mL and 1.20 × 10^−1^ mg/mL. Pabst et al. [13] determined the K_d_ values for three mAbs across current state of the art Protein A resins. mAbs 1 and 3 were of IgG1 isotype and mAb 2 was an IgG4 isotype. The reported K_d_ values ranged from 1.37 × 10^−3^ to 7.99 × 10^−3^ mg/mL for mAb1, 3.31 × 10^−3^ to 2.32 × 10^−2^ mg/mL for mab2, and 2.61 × 10^−3^ to 1.05 × 10^−2^ mg/mL for mab3.

**Figure 4:**
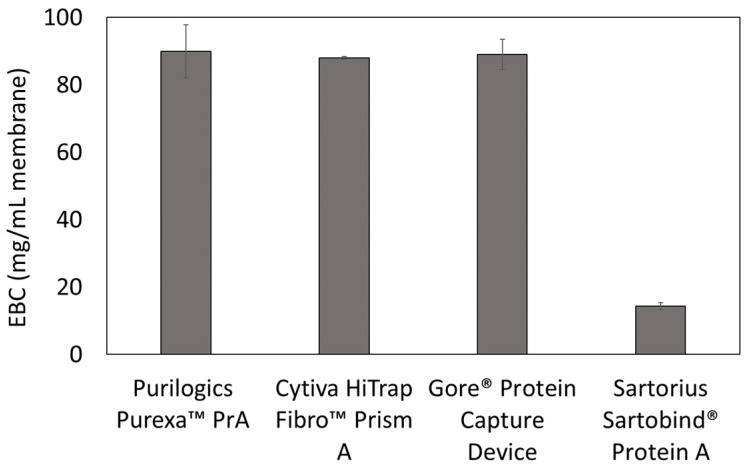
Equilibrium binding capacity (EBC) measurements at 120 s residence time. All membranes were loaded at 5 mg/mL hIgG in 1X PBS pH 7.4 buffer and eluted using 0.1 M citric acid pH 3 buffer.

Equilibrium binding capacity values also were measured in dynamic experiments. Figure 4 shows the equilibrium binding capacities at 5 mg/mL hIgG loading concentration. For the Purilogics Purexa™ PrA and Sartobind^®^ devices, the equilibrium binding capacities are not statistically different than the q_max_ measured in batch adsorption mode. Purilogics Purexa™ PrA, Cytiva HiTrap™ Fibro PrismA, and Gore^®^ Protein Capture Device have the highest capacities for Protein A membrane adsorbers reported to date. These three membranes achieve an hIgG EBC value of approximately 88 mg/mL, while the Sartorius Sartobind^®^ Protein A membrane reached 14 mg/mL.

There is a common belief that surface area is a key predictor of binding capacity. In this study, we tested that perception. Table 5 shows the specific surface area measurements of all Protein A devices. The measured specific surface areas did not have a strong correlation with the q_max_. The specific surface area of the Gore^®^ Protein Capture Device, 27.72 m^2^/g, is approximately 2.6 times higher than the Purilogics Purexa™ PrA device and 5.65 times greater than the Cytiva HiTrap™ Fibro PrismA device. Despite the large difference in specific surface area, these devices have similar q_max_ values, suggesting that specific surface area is not the key physical property impacting binding capacity. Other factors need to be considered, such as steric effects of Protein A ligand density [12], the IgG to Protein A binding stoichiometry after immobilization [12,34], Protein A immobilization chemistry [35], and reduction of Protein A activity during the immobilization procedure [36]. Evaluating these factors are beyond the scope and feasibility of this study.

The specific surface area of the Gore^®^ Protein Capture Device and the Cytiva HiTrap™ Fibro PrismA device highlight interesting features about the underlying support structure. The specific surface area of the Gore^®^ Protein Capture Device is comparable to the typical specific surface area of packed resin beds (30–40 m^2^/g) because both formats use porous beads. Where packed resin beds are constructed by simply packing a slurry of porous resin beads into a column, the Gore^®^ Protein Capture Device achieves high specific surface area by embedding porous silica beads into a PTFE matrix. According to the patent literature, the pore diameter of the Davisil silica beads embedded in the Gore^®^ Protein Capture Device is 100 nm with an estimated specific surface area of 40 m^2^/g (manufacturer information). For the Cytiva HiTrap™ Fibro PrismA, the specific surface area is about half of the Purilogics Purexa™ PrA membrane despite similar pore diameter. For this type of support, electrospinning is done to increase the available surface area to volume ratio. There is a common belief that electrospun fiber supports always offer higher surface areas than conventional macroporous membrane supports. However, the higher surface areas are achieved only at small pore diameters and reduced fiber diameter [37]. In other words, there is a direct correlation between fiber diameter and pore diameter. This would explain the lower specific surface area for the Cytiva HiTrap™ Fibro PrismA device.

### 3.3. Elution Behavior

**Figure 5:**
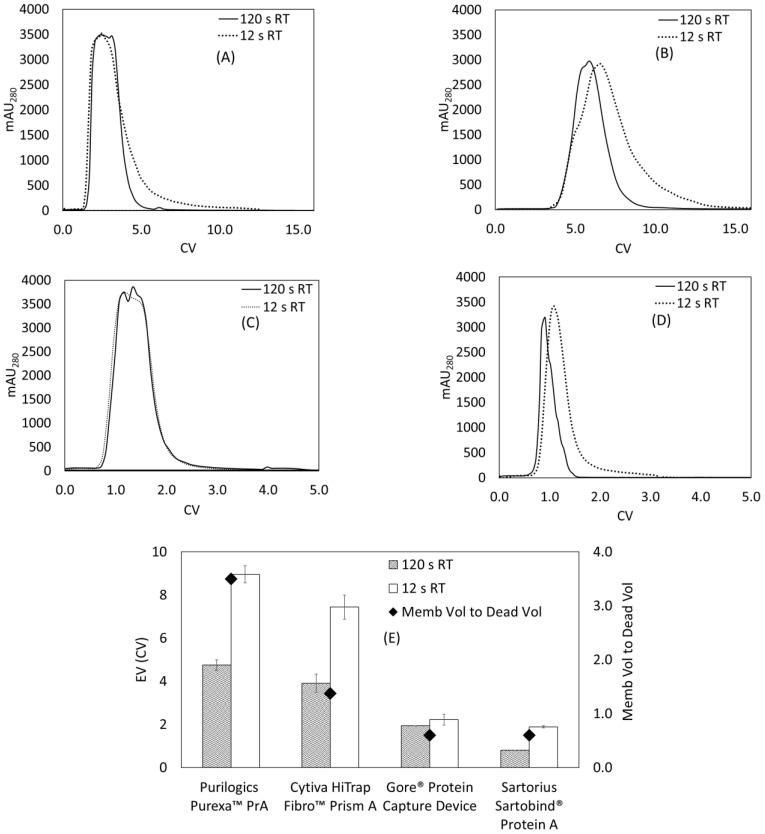
Elution behavior for Protein A membrane adsorbers at 12 and 120 s residence time. Membranes were saturated using 2 mg/mL hIgG in 1X PBS pH 7.4 buffer and eluted using 0.1 M citric acid pH 3 buffer. (**A**) Cytiva HiTrap Fibro™ PrismA, (**B**) Purilogics Purexa™ PrA, (**C**) Gore^®^ Protein Capture Device, (**D**) Sartorius Sartobind^®^ Protein A, (**E**) summary of measured EVs for all stationary phases at both flow rates, with the membrane to dead volume ratio graphed on the secondary *y*-axis.

Figure 5A–E shows the EVs for all membrane devices at 120 and 12 s residence times. The distinguishing feature of these elution curves is the increased tailing at shorter residence times. Table 6 shows the calculated tailing ratio using Equation (7) for all Protein A membranes. For all membranes, the tailing ratio is greater than one, which suggests that some degree of tailing occurs during the elution process. This makes sense as the elution is performed by a sharp drop in pH (to 3), which causes a sharp initial front of eluted protein. The tailing ratio for Purilogics Purexa™ PrA, Cytiva HiTrap Fibro™ Prism A, and Sartorius Sartobind^®^ Protein A show a significant increase at 12 s RT versus 120 s RT. For the Purilogics Purexa™ PrA, the elution curve at 12 s RT presents a shoulder that shifts the peak maximum to the right. This shift effectively reduces the tailing ratio by increasing the symmetry on both sides of the peak maximum. The Gore Protein Device does not show a significant difference in tailing behavior between 12 and 120 s RT.

A few additional observations can be made about the elution peak shapes. The elution curve of the Gore^®^ Protein Capture device, shown in Figure 5C, is flattened at the top. This is due to UV-VIS detector saturation, and has been observed in elution profiles of saturated Protein A resins [13]. There is a shoulder that appears in Figure 5B for the Purexa™ PrA 12 s RT elution curve. There is currently no explanation for this phenomenon.

For the Purilogics Purexa™ PrA, Cytiva HiTrap™ Fibro PrismA, and the Sartobind^®^ Protein A membranes, the EV approximately doubles as the residence time decreases from 120 to 12 s (Figure 5E). The effect of flow rate on elution curves is studied rarely, but a few papers have noted broadened elution peaks at higher flow rates in membrane adsorbers. Hardick [38] noted that weak anion-exchange electrospun nanofiber membranes, a similar format to the Cytiva HiTrap™ Fibro PrismA support, showed elution peak broadening as a function of flow rate. Boi et al. [33] found increased tailing as the elution flow rate increased in Sartobind^®^ Protein A membranes.

Peak tailing is tied to flow distribution and the specific format of the membrane adsorber device [39,40]. All membrane adsorbers evaluated in this study operate in direct-flow format. Madadkar et al. [41] evaluated the flow distribution in typical direct-flow (dead-end) membrane chromatography devices and concluded that the high aspect ratio (bed diameter to bed height) and the large dead volumes lead to poor fluid residence time distribution in the device. Poor flow distribution then leads to early breakthrough and peak broadening during elution, which is exacerbated at higher flow rates.

As predicted by that study, the device with the highest dead volume to membrane volume ratio, the Purilogics Purexa™ PrA membrane device, showed the highest EV (9.0 CVs at 12 s RT) followed by the Cytiva HiTrap™ Fibro PrismA membrane device (7.4 CVs at 12 s RT), and then the Sartobind^®^ device (1.9 at 12 s RT). The Gore^®^ Protein Capture Device showed low EVs that did not change with flow rate, suggesting that the Gore^®^ device has effective flow distribution. At 12 and 120 s RT, the Gore^®^ Protein Capture Device maintained an EV of 2.0 and 1.9 CV. By comparison, commercial Protein A resins show EVs between 1.8 and 3.8 CVs at 6 min residence time [13]. Importantly, the research-scale devices tested are all direct-flow columns. It is anticipated that commercial scale devices will use cassette formats with improved flow distribution properties.

One potential solution to increased tailing is to reduce the flow rate at elution for the membranes with high dead volume. As shown in Figure 5A–D, a reduction to 120 s residence time in the elution step led to reduction in EV by half for Purilogics Purexa™ PrA, Cytiva HiTrap™ Fibro PrismA and Sartorius Sartobind^®^ Protein A membranes. The reduction in EV is also mirrored by acetone-pulsing experiments where reduced tailing was observed at higher residence times (Figure S2 in Supplementary Materials).

Another alternative is to alter the membrane holder design to allow for a better flow distribution. Ghosh et al. [42] investigated one new device design where the incoming fluid flows laterally across the membrane stack, similar to crossflow filtration. Computational fluid models showed more uniform residence time distributions for the fluid [43]. Results from the models were supported by experimental results showing increased DBC_10_ and smaller EVs for a laterally fed membrane of the support, bed volume and dead volume to a direct-flow membrane chromatography device or a radial flow membrane chromatography device [41,44].

### 3.4. Clean-in-Place (CIP) Study

**Figure 6:**
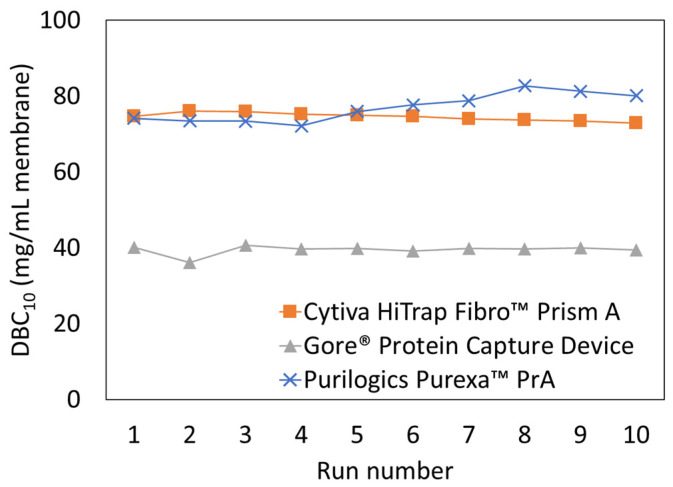
Dynamic binding capacity (DBC_10_) versus run number for three Protein A membrane adsorbers: Cytiva HiTrap Fibro™ PrismA, Purilogics Purexa™ PrA, and Gore^®^ Protein Capture Device. Two CVs of 0.1 M sodium hydroxide were used for CIP procedure after each run. All membrane adsorbers were loaded at 30 s residence time using 5 mg/mL hIgG in 1X PBS pH 7.4 buffer.

The effect of cleaning was evaluated for three high-performing membrane adsorbers: Purilogics Purexa™ PrA, Cytiva HiTrap™ Fibro PrismA, and Gore^®^ Protein Capture Device. The coefficient of variation for binding capacity was evaluated for each membrane to quantify the consistency of operation over 10 cycles. The Cytiva HiTrap™ Fibro PrismA had the lowest coefficient of variation (1.21) followed by the Gore^®^ Protein Capture Device (2.60) and then the Purilogics Purexa™ PrA device (3.61). The coefficient of variation indicates that the Cytiva HiTrap™ Fibro PrismA was the most consistent over the 10 runs but Figure 6 shows that the DBC_10_ slightly decreases over time. For Purilogics Purexa™ PrA, the coefficient of variation is higher but the membrane capacity increased over the 10 runs performed. The Gore^®^ Protein Capture Device had a low capacity during run 2 which had a significant effect on the coefficient of variation over the 10 runs. Based on this limited cycle study using hIgG, all membranes maintained performance over 10 runs when using 0.1 M NaOH as the CIP agent. This study did not evaluate how the performance may change over time when binding a target protein from a cell culture supernatant that contains process impurities due to limited availability of cell culture supernatant.

### 3.5. Permeability and Pressure Drop

**Figure 7:**
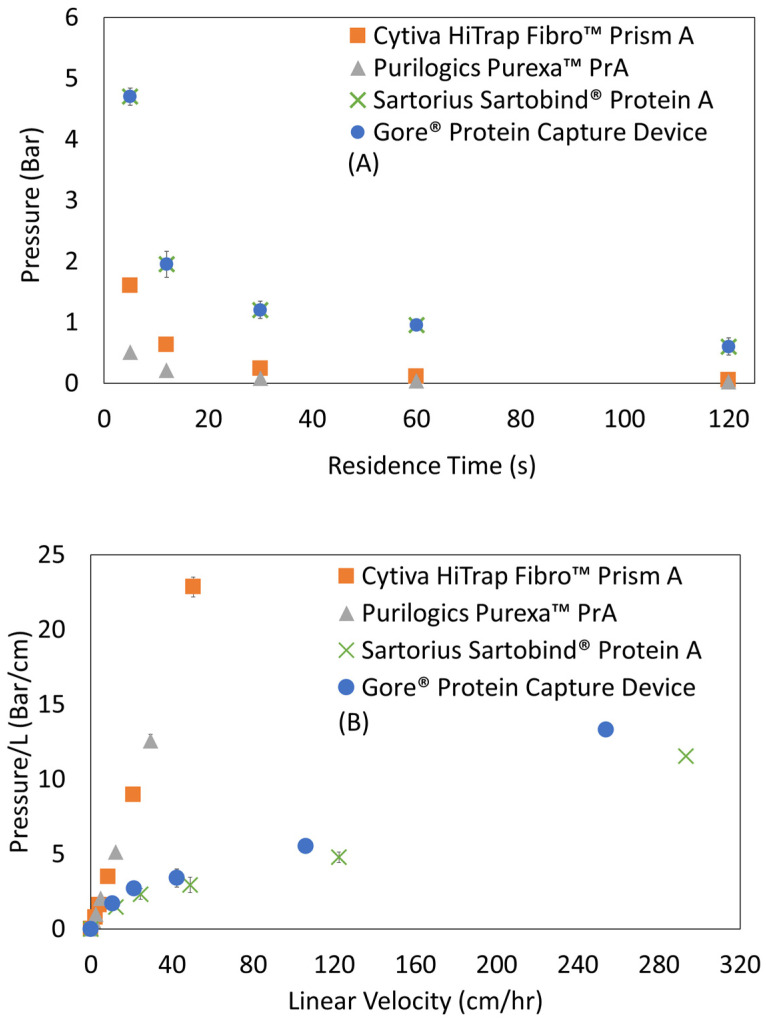
Pressure profiles of Protein A membrane adsorbers. (**A**) Absolute pressure versus residence time. (**B**) Absolute pressure normalized by membrane bed thickness versus linear velocity, where the slope is interpreted as permeability^−1^. Permeability values for all membrane adsorbers are given in Table 5.

Figure 7A shows absolute pressure drop versus residence time for Protein A membranes. The absolute pressure drop values of these research scale devices are low, remaining below 2 bar at residence times as short as 12 s.

The pressure profile for resin columns typically is characterized by graphing the pressure drop normalized by bed height versus the linear velocity, and the permeability typically is calculated from the slope using Darcy’s Law. For this study, Figure 7B shows pressure drop per thickness versus linear velocity for membrane adsorbers, and Table 5 presents the calculated permeabilities. Permeabilities measured in this study ranged from 0.62 × 10^−15^ m^2^ to 6.19 × 10^−15^ m^2^, with Cytiva HiTrap™ Fibro PrismA being the lowest permeability device followed by Purilogics Purexa™ PrA, Gore^®^ Protein Capture Device, and Sartorius Sartobind^®^ Protein A. Lower permeabilities were observed for smaller pore diameter membranes (Table 5). Comparatively, Protein A resin permeabilities, calculated from pressure profiles given in a recent study by Pabst et al., are in the range from 4.27 × 10^−12^ m^2^ to 1.22 × 10^−12^ m^2^.

Comparing the permeabilities of resins versus membranes, Protein A resin bed permeabilities are higher than Protein A membranes. This finding is surprising due to the typical flow profile of Protein A membranes versus resins. Protein A membranes are typically operated at seconds of residence time where their absolute pressure drop is still low (<2 bar at residence time of 10 s). On the other hand, resins are operated at minutes of residence time because higher flow rates result in even higher pressure drops due to bed compaction. For comparison, a typical research-scale protein A resin having 10 cm bed height shows 3–4 bar of absolute pressure drop at 120 s residence time, whereas the Protein A membranes in this study with bed heights as high as 0.4 cm show less than 0.6 bar of absolute pressure drop at the same residence time/linear velocity [13]. Two important conclusions can be drawn from the discrepancy between absolute pressure drops and permeabilities. Firstly, that the higher absolute pressure drop found in Protein A resins is a function of the bed height rather than the permeability of the resin bed itself. This conclusion agrees with a study by Herigstad et al. that showed that the permeability of a monolith, 5.74 × 10^−15^ m^2^, was lower than that of MabSelect, 9.5 × 10^−12^ m^2^ [15], despite the lower absolute pressure drop for the monolith bed. Secondly, for membranes, a lower absolute pressure drop is a function of shorter bed height rather than the permeability of the material; and, even more importantly, the ability to operate at seconds of residence time despite lower permeability is due to the device format, i.e., a large pore structure and an aspect ratio with large diameter to bed height.

This important effect of device format is also highlighted by the unique nature of the composite Gore^®^ Protein Capture Device. It embeds silica beads in the PTFE matrix in a way that does not allow compression at high flow rates. The use of silica beads themselves adds rigidity, as it is harder than materials like agarose and polymethacrylate, and increases surface area for immobilization and binding [45]. The resulting device is a composite with a flow profile that allows for lower back pressure at seconds of residence time (where resin beds cannot operate) but a binding profile like a resin bed, as it is flow dependent.

### 3.6. Capture from Clarified Cell Culture Harvest

**Figure 8:**
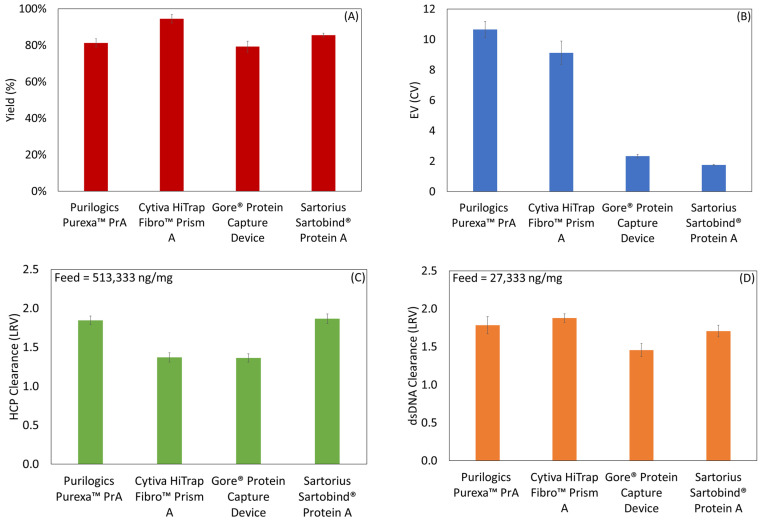
Process metrics for Protein A membrane adsorbers loaded with IgG1 mAb from cell culture supernatant. (**A**) Yield, (**B**) EV, (**C**) HCP clearance, and (**D**) dsDNA clearance.

Membranes were challenged by purifying an IgG1 mAb directly from clarified cell culture supernatant. The mAb concentration was 0.9 mg/mL as measured by a Cedex Bioanalyzer. Figure 8A–D shows the results of the process metrics: yield, EV, HCP clearance and dsDNA clearance. All devices had yields at or above 80%. Measured EVs using mAb followed the same trend as using the model hIgG. HCP LRVs were between 1.37 and 1.87 from a feed that had 513,333 ± 62,186 ng/mg mAb. Using EV and yield, final HCP concentrations (in ng HCP/mg mAb) were calculated to be 1476 ± 108 for Purexa™ PrA, 3385 ± 372 for Cytiva HiTrap™ Fibro PrismA, 4373 ± 287 for Gore^®^ Protein Capture Device, and 3182 ± 243 for Sartorius Sartorbind^®^ Protein A. The dsDNA LRVs were between 1.46 and 1.78 from a starting concentration of 27,333 ± 3456 ng/mg mAb. According to FDA regulations, the final product should contain less than 100 pg of host cell DNA per dose and 100 ppm of HCP per mg of antibody product [46,47]. High clearance in the Protein A step is critical towards meeting those criteria.

Evaluating all metrics comparatively, no one membrane performs best in all categories. The Gore^®^ Protein Capture Device has a low EV but is inferior to Purilogics Purexa™ PrA, Cytiva HiTrap™ Fibro PrismA, and Sartorius Sartorbind^®^ Protein A in all other metrics. The Cytiva HiTrap™ Fibro PrismA membrane has the highest yield but is inferior to the Purilogics Purexa™ PrA, and Sartorius Sartobind^®^ Protein A devices in HCP clearance, and has poorer EV than Gore^®^ Protein Capture Device and Sartorius Sartobind^®^ Protein A. The Purilogics Purexa™ PrA and Sartorius Sartobind^®^ Protein A membranes have the best HCP clearance but the Purilogics Purexa™ PrA membrane has the highest EV and the Sartorius Sartobind^®^ Protein has the lowest binding capacity, which means that each run results in about 85% less mass of mAb processed compared to the Purilogics Purexa™ PrA, and Cytiva HiTrap™ Fibro PrismA devices.

Outside of EV performance, which is tied to dead volume and flow distribution (as previously discussed), the yield and impurity clearance metrics cannot be linked directly to the measured physical characteristics of each device. Nevertheless, the yield and overall clearance capacity of the membrane devices can be compared to typical Protein A resin columns. According to a recent study, yields from current state-of-the-art Protein A resins are between 80 and 90%. HCP and DNA LRVs can vary widely depending on the molecule and the use of in-house assays versus generic commercial HCP kits. In this study, the HCP and DNA kit used were generic and may result in lower values. In the cited study, in-house assays with antibodies raised against the HCP from their CHO cell lines were used, and the resulting HCP and DNA LRVs were between 2 and 3 and 2.5 and 3.5 respectively [13].

## 4. New Membrane Product

During the course of this study, a new Sartorius Sartobind^®^ Protein A membrane was developed and released. This device was not available for purchase at the time of our study. This new device, Convecdiff Protein A, utilizes a mix of convection and diffusion as the transport mechanism resulting in flow-dependent binding. The range of binding capacities is from 35.2 mg/mL at 6 s residence time to 50 mg/mL at 2 min RT. EVs range from 4.4 to 5.1 CV and yield from 98 to 99.4%. The devices also show similar performance from 1.2 to 70 mL device volume [23].

## 5. Conclusions

For the first time in the open literature, commercial Protein A membranes have been comparatively characterized on the basis of performance and physical properties. Evaluating the performance of these devices in the context of their physical properties gave insight into differences among them and more generally, between Protein A membranes and resins. Firstly, it was discovered that specific surface area is not the key predictor of maximum binding capacity for these membranes; Purilogics Purexa™ PrA, Gore^®^ Protein Capture Device, and Cytiva HiTrap Fibro™ PrismA and all reach nearly 90 mg/mL hIgG capacity despite large specific surface area differences. In terms of dynamic binding capacity, Purilogics Purexa™ PrA and Cytiva HiTrap Fibro™ PrismA show flow rate-independent binding from 5 to 60 s of residence time, reaching ~70 mg/mL at 5 s RT. This performance is unprecedented and illustrates how capture membranes vastly outperform resins in speed without sacrificing binding capacity.

Unlike the other membrane devices, the binding capacity of Gore^®^ Protein Capture Device varied with flow rate, suggesting that it operates via a diffusion-limited mechanism of transport similar to resin beads. This behavior was attributed to the composite membrane/particle structure of the media. Nevertheless, the permeability and flow distribution properties of this silica–PTFE composite device yielded low EVs and flow-independent EVs.

For studies performed with the cell culture supernatant feed, the yield, HCP and DNA clearance values are within the range found in the literature and acceptable for industrial purification processes. However, HCP and DNA clearance may be better predicted with in-house assays prepared against cell lines expressing that particular biomolecule.

The notable results from this study can serve as a guide for research and development scientists and engineers to understand the benefits and limitations of these novel devices and how Protein A membrane chromatography can be used in the context of their specific processes to meet their particular needs.

The next frontiers of Protein A membrane development and affinity membrane development will include: (i) improving device designs with lower dead volume and better flow distribution to reduce EV, (ii) producing and evaluating membrane devices at process scale, (iii) understanding process performance when using membrane adsorbers in continuous processes, and (iv) characterizing performance with larger modalities where flow-dependent binding in resins may result in lower binding capacity and process productivity compared to membranes. The results shown here represent a step toward standardization of the use of Protein A membrane chromatography in the protein purification industry.

## Figures and Tables

**Table 1:** Chromatography procedure for bind and elute studies on Protein A stationary phases.

Chromatography Step	Buffer	Volume Used
Equilibrate	1X PBS pH 7.4 (B1)	20 CVs
Load	hIgG in 1X PBS pH 7.4	varies
Wash	1X PBS pH 7.4 (B1)	5 mL
Elute	0.1 M citric acid pH 3.0 (E1)	varies
CIP	0.1 M NaOH	2 CVs

**Table 2:** Flow rate versus residence time for all Protein A stationary phases.

RT (s)	Flow Rate (mL/min)			
	Purilogics Purexa™ PrA	Gore^®^ Protein Capture Device	Cytiva HiTrap Fibro™ PrismA	Sartorius Sartobind^®^ Protein A
120	0.1	0.5	0.2	1.0
60	0.2	1.0	0.4	2.0
30	0.4	2.0	0.8	4.0
12	1.0	5.0	2.0	10.0
5	2.4	12.0	4.8	24.0

**Table 3:** Biophysical characterization of monoclonal antibody used in this study.

Name	Isotype	Isoelectric Point	Molecular Weight (g/mol)
Anti-HIV mAb	IgG1	8.42 *	154,475 *

* Predicted based on amino acid sequence.

**Table 4:** Langmuir adsorption isotherm parameters for hIgG static adsorption onto Protein A stationary phases.

	Fitted Parameters		Statistical Metrics
	q_max_ (mg/mL)	K_d_ (mg/mL)	r^2^
Purilogics Purexa™ PrA	83.95 ± 3.44	7.07 × 10^−2^ ± 1.80 × 10^−2^	0.95
Sartorius Sartobind^®^ Protein A	14.23 ± 0.63	7.63 × 10^−2^ ± 2.48 × 10^−2^	0.91

**Table 5:** Physical properties of all Protein A stationary phases.

Membrane	Commercial Membrane Matrix	Memb. Vol (mL)	Thickness (mm)	Permeability (m^2^ × 10^−15^)	Specific Surface Area (m^2^/g)	Pore Diameter (um)	
						Measured	Manufacturer
Purilogics Purexa™ PrA	Regenerated cellulose	0.2	0.4	0.65	10.54 ± 0.33	0.28 ± 0.05	0.20
Cytiva HiTrap Fibro™ Prism A	Derivatized electrospun cellulose	0.4	0.7	0.62	6.23 ± 0.97	0.25 ± 0.03	n.a.
Sartorius Sartobind^®^ Protein A	Stabilized cellulose	2.0	4.0	6.91	4.90 ± 0.17	0.37 ± 0.03	0.45
Gore^®^ Protein Device	porous silica in expanded polytetrafluoroethylene	1.0	3.5	5.14	27.72 ± 0.61	n.a.	0.1 *

* Sourced from the patent literature.

**Table 6:** Tailing ratio of Protein A membrane adsorbers at 12 s and 120 s RT.

	Tailing Ratio (T)	
	12 s RT	120 s RT
Purilogics Purexa™ PrA	1.56 ± 0.07	1.16 ± 0.06
Cytiva HiTrap Fibro™ Prism A	3.28 ± 0.33	1.18 ± 0.13
Gore^®^ Protein Device	1.50 ± 0.03	1.53 ± 0.02
Sartorius Sartobind^®^ Protein A	2.52 ± 0.09	1.45 ± 0.04

## Data Availability

The data required to reproduce these findings are available by request to the corresponding author.

## Supplementary Materials

The following supporting information can be downloaded at: https://www.mdpi.com/article/10.3390/membranes13050511/s1, Figure S1. Binding kinetics curves for Protein A membrane adsorbers. Figure S2. Acetone tracer curves as a function of residence time.
