# Supplementary material for: Comparative Evaluation of Commercial Protein A Membranes for the Rapid Purification of Antibodies
Source: Membranes (Basel). 2023 May 12;13(5):511. doi: 10.3390/membranes13050511 (PMC10220532; doi:10.3390/membranes13050511)
Supplement: Supplementary file 1 [file membranes-13-00511-s001.zip › membranes-2368349-supplementary.pdf]

membranes

Supplementary Materials

Comparative Evaluation of Commercial Protein A Membranes for the Rapid Purification of Antibodies

Joshua Osuofa and Scott M. Husson *

Department of Chemical and Biomolecular Engineering, Clemson University, Clemson, SC 29634, USA
* Correspondence: shusson@clemson.edu

Contents

Figure S1. Binding kinetics curves for Protein A membrane adsorbers. Membranes were soaked in 5 mg/mL hIgG protein solution in 1x PBS pH 7.4 buffer and set on a shaker (r.t., 120 rpm) to reach equilibrium. The protein concentration was monitored using a Thermo Fisher Nanodrop One and the static binding capacity (SBC) at each time point was calculated using the difference in initial protein concentration and protein concentration at that time point. (A) Sartobind® Protein A, (B) Purilogics Purexa™ PrA, (C) Gore® Protein Capture Device.

Figure S2. Acetone tracer curves as a function of residence time. Acetone with volume equal to 5% of each membrane column volume (CV) was injected at two different residence times, 120 and 12 s: (A) Cytiva HiTrap Fibro™ PrismA, (B) Purilogics Purexa™ PrA, (C) Gore® Protein Device, (D) Sartorius Sartobind® Protein A.

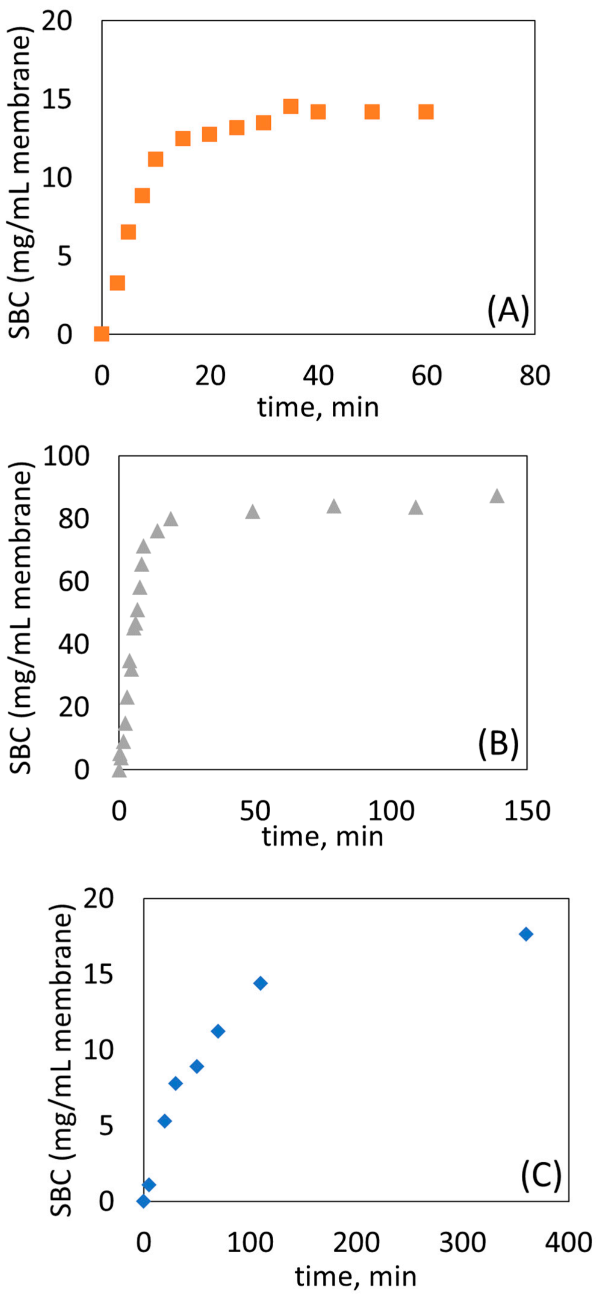

Figure S1. Binding kinetics curves for Protein A membrane adsorbers. Membranes were soaked in 5 mg/mL hIgG protein solution in 1× PBS pH 7.4 buffer and set on a shaker (r.t., 120 rpm) to reach equilibrium. The protein concentration was monitored using a Thermo Fisher Nanodrop One and the static binding capacity (SBC) at each time point was calculated using the difference in initial protein concentration and protein concentration at that time point. (**A**) Sartobind® Protein A, (**B**) Purilogics Purexa™ PrA, (**C**) Gore® Protein Capture Device.

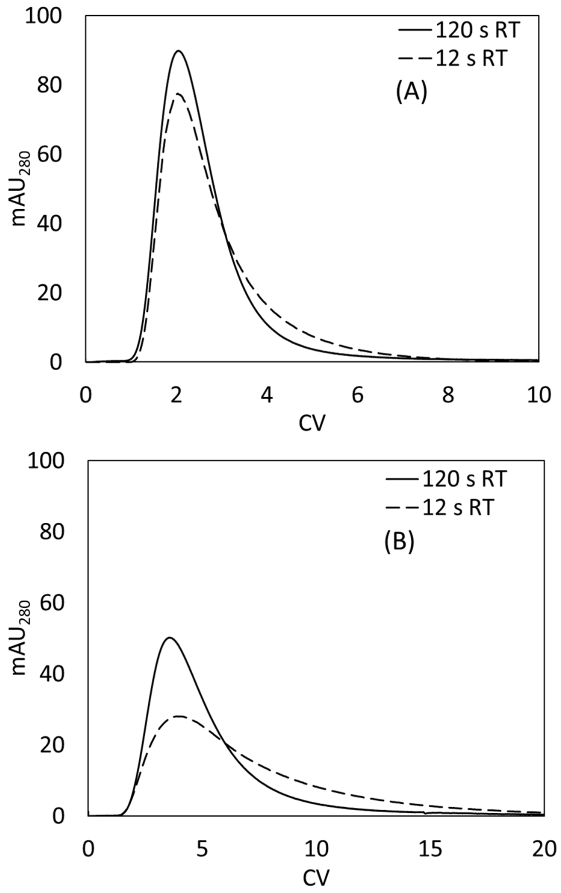
120 s RT
12 s RT
(A)
mAU280
CV
120 s RT
12 s RT
(B)
mAU280
CV

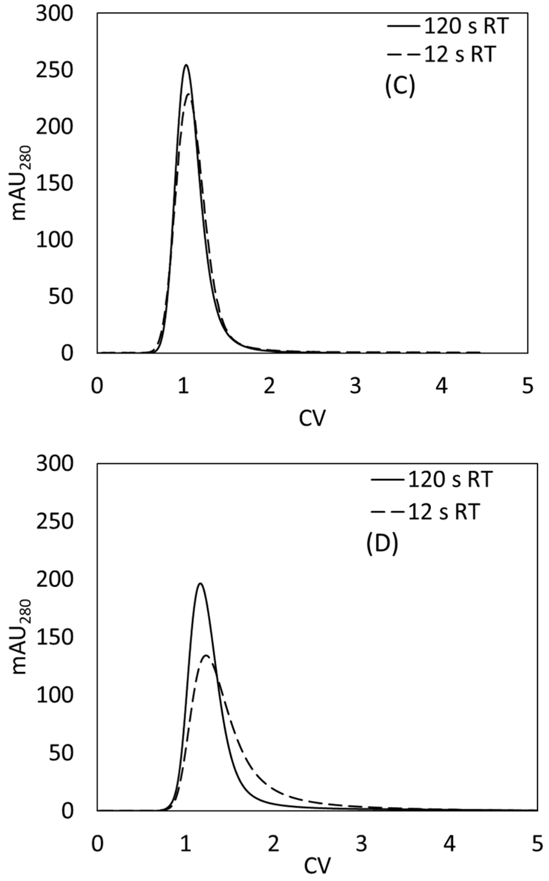

Figure S2. Acetone tracer curves as a function of residence time. Acetone with volume equal to 5% of each membrane column volume (CV) was injected at two different residence times, 120 and 12 s: (**A**) Cytiva HiTrap Fibro™ PrismA, (**B**) Purilogics Purexa™ PrA, (**C**) Gore® Protein Device, (**D**) Sartorius Sartobind® Protein A.
